# Editorial: The relationship between diabetes and cancers and its underlying mechanisms, volume II

**DOI:** 10.3389/fendo.2023.1357577

**Published:** 2024-01-08

**Authors:** Qiang Huo, Shuo Wang, Ying Hou, Reginald M. Gorczynski, Yining Shen, Bin Wang, Hanyi Ge, Tao Li

**Affiliations:** ^1^Clinical Research Center, Nanjing Jiangbei Hospital, Nanjing, China; ^2^Center for Translational Medicine, Zibo Central Hospital, Zibo, China; ^3^Department of Oncology, Shibo High-Tech Hospital, Zibo, China; ^4^Department of Otolaryngology, Zibo Central Hospital, Zibo, China; ^5^Department of Surgery & Immunology, University of Toronto, Toronto, ON, Canada; ^6^College of Integrated Traditional Chinese and Western Medicine, Jining Medical University, Jining, China; ^7^Department of Oncology, Zibo First Hospital, Zibo, China; ^8^Department of Stomatology, Zibo Central Hospital, Zibo, China

**Keywords:** diabetes mellitus, type-2 diabetes, cancer morbidity and mortality, oral cancer, obesity

Diabetes mellitus (DM), as well as cancer, has been recognized as one of the major life-threatening diseases that caused increased health care costs, deteriorated quality of life, and premature death (1, 2). The morbidity of diabetes and various cancers has been increasing around the globe, suggesting that there may be a potential relationship between DM and cancer morbidity or mortality (3, 4). Moreover, the association of DM with oral conditions has been studied for years (5, 6), and recent studies have demonstrated the possible linkage between DM and oral cancer (7, 8). Current evidence has been evaluated by a systematic review and meta-analysis which indicated that patients with DM have a higher risk in developing oral cancer and oral potentially malignant disorders (OPMD), and patients with oral cancer suffering from DM have a higher mortality (7). Retrospective research also confirmed an association between glucose metabolism disorder (GMD) and oral cancer, by screening 573 patients who had undergone maxillofacial surgery under general anesthesia (8).

Our last collections on this Research Topic have advanced current knowledge on the clinical or biological mechanisms underlying the association of DM with cancer development in a variety of study protocols or article types, including original research, review, and case report (9). During this collecting, we emphasized more on original works, and have reviewed and collected excellent original research from different regions, such as those from Finland, Greece, and China.

Evidence from recent literature has indicated that type 2 diabetes (T2D) is associated with the morbidity and/or mortality of several cancers. In the open-cohort study assessing the association of obesity and T2D with cancer incidence and survival, Sifaki-Pistolla et al. analyzed the data from the Cancer Registry of Crete in their final sample of 49,256 cancer patients, and demonstrated that people with T2D or obesity had higher risk of developing kidney, breast, colorectal, endometrial, esophageal, pancreatic and liver cancer, and both obesity and diabetes in cancer patients presented a strong association with poor survival. Their findings also indicated that health care interventions aiming to reduce the burden of T2D and obesity should be involved in cancer management.

Peng et al. also investigated the relationship between diabetes and cancer incidence as well as mortality in the UK Biobank prospective cohort, but they focused more on patients with different ages for the diagnosis of diabetes. The authors summarized data from a total of 26,318 diabetics and 105,272 controls matched by the same baseline age, and used Cox proportional hazard model to examine the associations of diabetes at different diagnostic ages with cancer incidence and mortality. Their findings showed that participants with diabetes diagnosed at 51–60 years were correlated with increased morbidity and mortality of various site-specific cancers, suggesting that the age at diagnosis of T2D, as well as tobacco control, is important for cancer management.

On the other hand, Huo et al. estimated the association between T2D and hepatocellular carcinoma (HCC) in East Asian populations using bidirectional Mendelian randomization (MR) analyses and adopting summary statistics from genome-wide association studies (GWAS) related to T2D and HCC. Their analyses supported the inverse association between T2D and HCC in East Asian populations, with or without the adjustment for potential confounders including chronic hepatitis, body mass index (BMI), and alcohol intake frequency, and suggested an important clinical implication for the prevention and management of these conditions.

The burden of pharmacologically treated diabetes differs around the world, especially among different socioeconomic groups. Guzman-Castillo et al. developed and validated a multi-state life table model by analyzing Finnish total population data for those aged 30 years on T2D medication and mortality, suggesting that the lowest income group could expect more rapid increases in the number with T2D compared to the highest income group, and the number of years lived without T2D could decrease up to 6 years for men in the lowest income group. Their evidence-based forecasting model predicted that both the number of people living with T2D and associated life expectancy with T2D will increase over the next two decades accompanied by an increase in inequalities among different socioeconomic groups, which emphasizes the coming societal challenge and urgent need for effective prevention.

Though in vitro and animal studies may suggest a beneficial effect of rosiglitazone on prostate cancer cells, Tseng investigated the risk of prostate cancer in in male patients with T2D in Taiwan, using the database of the National Health Insurance. This study enrolled a total of 11,495 ever users and 11,495 never users of rosiglitazone matched on propensity score, with follow-up information over six years. The author estimated the effect of rosiglitazone on prostate cancer risk by Cox proportional hazard model incorporated with the inverse probability of treatment weighting using the propensity score, and found that rosiglitazone has a null effect on the risk of prostate cancer. Though rosiglitazone has been used as an antidiabetic drug to treat hyperglycemia in patients with T2D by improving insulin resistance via activation of peroxisome proliferator-activator receptor gamma (PPARγ), this study suggests that previous results derived from cellular studies should be carefully interpreted and clinical trials in humans are pivotal to elucidate the roles of rosiglitazone as well as other thiazolidinedione (TZD) compounds in the development or prevention of prostate cancer.

The aims and objectives of this Research Topic are to collect laboratorial, clinical and epidemiological evidence, and constructive views, comments, and hypotheses related to the association between diabetes and cancer-specific incidence and/or survival. Based on the current evidence collected in our Research Topic, laboratory findings should be carefully interpreted and clinical trials in humans are pivotal to elucidate these roles. Meanwhile, cancer screening in patients with DM, or diabetes screening in cancer patients should be emphasized and extensively promoted, as well as precautions or preventive intervention among these individuals.

## Author contributions

QH: Data curation, Formal Analysis, Project administration, Resources, Supervision, Writing – original draft. SW: Investigation, Methodology, Validation, Writing – original draft. YH: Data curation, Formal Analysis, Validation, Writing – review & editing. RG: Methodology, Project administration, Writing – review & editing. YS: Data curation, Project administration, Writing – review & editing. BW: Conceptualization, Supervision, Writing – review & editing. HG: Project administration, Supervision, Writing – review & editing. TL: Conceptualization, Supervision, Writing – review & editing.
